# Neural perspectives on morality due to beguiling mechanisms

**DOI:** 10.3389/fpsyg.2023.1151155

**Published:** 2024-01-31

**Authors:** Haavard Koppang, Søren Wenstøp, Jaime A. Pineda

**Affiliations:** ^1^BI Norwegian Business School, Oslo, Norway; ^2^Department of Economics, Innovation and Society, Østfold University College, Halden, Østfold, Norway; ^3^University of California, San Diego, San Diego, CA, United States

**Keywords:** neural perspectives, deception and charm, beguiling mechanisms, mindsets, morality, hierarchical-heterarchical processes

## Abstract

We consider deception an example of behavior that challenges traditional explanations of moral behavior. Beguiling mechanisms, by which we mean deceptiveness with charming seduction for diversion, subtly influence moral sensitivity and judgment in moral dilemma situations. The duality of beguiling mechanisms is important to grasp, including how they relate to the ambiguity of situations. Further, we view moral behavior as quasi-adaptive, affectively based, and reliant on the processes of social cognition, arising out of a set of domain-general primitive predispositions that aggregate to produce moral “mindsets” and increasingly complex moral actions. Building on recent theoretical developments, contend that morality involves a complex heterarchical-hierarchical neurological architecture, where activity is dynamically and contextually dependent, as well as dependent on evolved brain structures and early life year socialization. We contribute to conceptualizing moral behavior from an integrated modern neural perspective. This provides a balance between moral decisions as situational, emotional, and genetically completed non-conscious processes, and the more traditional view of conscious reasoning. Beguiling mechanisms illustrate an integrative model of morality, consistent with emerging insights from affective and cognitive neuroscience.

## 1 Introduction

Mental processes, including moral and emotional thinking, follow a continuum, from controlled to automatic (Bargh, 1994). However, the view currently emerging from cognitive neuroscience, which acknowledges that the brain is a network of highly interconnected parts, suggests there is a greater, richer, and more complex continuum than previously understood. We should note that a multiplicity of automatic brain processes operating in the dark, outside conscious awareness, will always undergird even the most controlled, deliberate, and explicit forms of mental labor.

Some of these implicit, unconscious processes pick up informational cues and from the external context, others build on information from the inner milieu of the body. Bargh (2017) presents several experiments showing that even small and trivial contextual factors can alter the social attitudes of moral actors without their awareness, in ways that have behavioral implications. Emotions, meanwhile, respond both to cues in the context and to the needs of the biophysical system of the body (Damasio, 2021, Ch. 3). They give rise to distinct feelings that play a central role in guiding social and moral behavior (Solms, 2021, Ch. 5).

We argue that some of the most influential theories on moral behavior, from Piaget (1932) and Kohlberg (1969), rest on assumptions at odds with the body of evidence from affective, social, and cognitive neuroscience. Hence, a morality grounded in modern psychology, biology, neuroscience, and culture presents a challenge to traditional cross-disciplinary research (Haidt, 2013). In this paper, we describe a balance between moral mindsets and moral decisions as situational, emotional, and largely sub-conscious. This contrasts the position represented by the more traditional view of psychology and philosophy that conscious deliberative reasoning is decisive.

Our goal is to increase awareness of moral agents facing complex situational tradeoffs between reason and emotion, without being absorbed by an analytical and calculative process that might promote unethical behavior (Zhong, 2011). Recent developments in research on ethical decisions show that deliberative decision-making leads to increased unethical behaviors (Zhong, 2011) by circumventing areas of intuitive emotional discomfort. Deliberative decision-making, including a calculative mindset, appears to impair an individual’s capability to deal with moral dilemmas, making them more rather than less likely to engage in unethical behaviors most of the time. Our perspective is that emotional discomfort is an important resource for moral decision-making, broadly because it serves as a fairly reliable indicator that something is not on the right track^1^.

We do not claim there are “real” and “objective” solutions to moral dilemmas provided by any one simple mechanism, nor that our answers depend on them. By the term “mechanism” we refrain from a mechanistic framework that explains the natural world in terms of mechanical processes, including human behavior (Bechtel, 2008, on R. Descartes). We use the term “mechanism” to refer to mental mechanisms that are not naïve or simplistic accounts. Thus, human freedom and dignity are complex because of mechanisms that change and evolve as a function of experience (Bechtel, 2008, p. 240). By “mechanism” we do not refer to laws that, without exceptions, are precise as to necessary and sufficient conditions for when various mechanisms come online. Rather, we normally cannot state necessary and sufficient conditions for when and how various neural mechanisms activate. Our illustrations also show that the mind/brain system is a dynamically changing system that must function both as affective and reasoning one.

We focus our argument on beguiling mechanisms as an example of how subtle factors can play a significant role by generating alternative mindsets, explained metaphorically by dual process psychological theory. We consider a type of deception, a kind of deceptiveness with charming seduction for diversion^2^, as something that provides a challenge for ethics, a challenge that is addressed through neural inspired models.

Even though there is an interesting relationship between ethics and deception, including morality and personality, we limit our work to a general approach rather than more specific approaches as, e.g., The Dark Triad traits and moral and social values – characterized by *narcissism* (*cf.* vanity and self-centeredness), *Machiavellianism* (*cf.* manipulation and cynicism) and *psychopathy* (*cf.* callous and social attitudes and impulsivity). A background for this demarcation is our general orientation on neural perspectives to challenge traditional perspectives on morality due to beguiling mechanisms, below the level of conscious awareness.

Hence, the role of intention and commitments to normative ethical theory, standard utilitarianism, and deontology, are not central aspects of our argument. We take most of the psychological interaction to take place below the level of reflective conscious awareness. The moral mind is not transparent to itself in the manner traditionally assumed. Some of the important decision systems involved are inherently implicit and “unconscious”; the manner, for example, in which the invisible hands of *associations* lead us (Redish, 2013, 65–74) and *habits* (Wood, 2019: Part 2; Redish, 2013, 87–96) to arrive at certain conclusions rather than others in the implicit tree of choices. Agents, in this view, never act on explicit conscious deliberation in isolation, but contextually, within a range of implicit mental processes and multiple interacting decision-systems.

Finally, we do not intend to provide an exhaustive taxonomy of “beguiling mechanisms” and their role in moral behavior. Our goal is simply to present a sketch, particularly one that emphasizes how we sometimes divert attention from prejudices, disturbing feelings, or calculative mindsets, and where the charming and persuasive presentation of an alternative or even mindset is included.

The relationship between “rational,” calculative mind (more left-brain hemisphere) and intuitive processes (more right-brain hemisphere) in moral decision making is one of the many mysteries that characterize humans. Zhong (2011) claims that people are intuitively ethical, pro-social, and cooperative, unless they take time to deliberate, which leads them to be more self-interested and immoral. Evidence from human neuroscience supports this position (Pfaff, 2015), as does work in primatology (Waal, 1996, 2016). This is relevant given the close resemblance between the sociality of humans and other primates, and the developmental psychology which suggests early onset of pro-social attitudes in children (Narvaez, 2014; Gopnik, 2016).

Wang et al. (2014) explore the ethical and social consequences of a calculative mindset, suggesting that calculation may be a special deliberation. In Rand (2016) “intuitive” is framed within economic games and entails strategies that are successful in daily life and become automatized as intuitions. Zhong and Haidt repeatedly use the combination of terms such as “moral intuition.” In Rand and Kraft-Todd (2014) “intuitive mindset” occurs, and by activating that mindset, there is more cooperation than when switching on a “deliberative mindset.” Now, we do not believe that mindset and changing of it leads to a shift in automatic intuition.

We assume that automatic intuition is stable, whereas situations that influence or manipulate intuition vary as an effect of *in situ* (in original or natural location) factors. In this view, different types of situations can trigger different mindsets rooted in different underlying neurological systems. This gives rise to the characteristic contrasting mindsets we see in human morality. There are those concerned with competition and preservation and safety (the complex of proto-reptilian emotions fight, freeze, flee, faint) on the one hand. And those geared toward cooperation and engagement (associated with the mammalian emotions care and play) on the other (Narvaez, 2014). We conceive beguiling mechanisms is conceived as involving multiple, sometimes competing, brain systems that negotiate moral interactions.

## 2 Heterarchical-hierarchical neurological architecture for beguiling mechanisms

A range of studies using diverse methods support a metaphorically dual-process theory of moral judgment to account for ethical behaviors. According to this, controlled cognitive processes enable utilitarian moral judgments (allegedly favoring the “greater good” over individual rights), while deontological judgments (allegedly favoring individual rights) are driven more by intuitive emotional responses (Brand, 2016). Kahane et al. (2012), Koenigs et al. (2007) and others associate utilitarian judgments with longer response times, and with increased activation in the dorsolateral and ventromedial prefrontal cortex, and inferior parietal lobe (based on fMRI imaging techniques). These are areas implicated in deliberative processing. In contrast, it associates deontological judgments with greater activation in areas related to higher-order affective processing, such as the ventromedial prefrontal cortex, the superior temporal sulcus, and the amygdala (Greene et al., 2001).

Emotional regions of interest, including the medial frontal gyrus, posterior cingulate gyrus, and angular gyrus, are more active when subjects solve moral dilemmas that involve personal as opposed to impersonal actions. Several studies have suggested that emotions play an integral role in moral judgments (Haidt, 2001; Greene et al., 2004; Moll et al., 2007; Greene et al., 2008). It should be noted, however, that the fundamental affective structures and rudimentary emotional qualities represented as feelings are all sub-cortically based (Panksepp, 1998; Solms, 2021). The emotions that play a part in moral judgments, even as specific cortical structures are involved in important ways, originate sub-cortical brain activity, most notably in the upper brain stem (Solms, 2021). Cortical activity can sometimes indicate further treatment, or even rationalizations, of moral decisions that are already made or prepared subcortically.

In contrast to moral dilemmas, impersonal dilemmas, selectively engage areas associated with working memory, such as regions of the middle frontal gyrus and posterior parietal cortex. Presumably, these regions support the abstract reasoning necessary to weigh the benefits of the courses of action. We have interpreted these various neural activations to reflect unique neural systems that underlie utilitarian and deontological moral judgments, not only in extreme dilemmas. We argue that this may be true as a first approximation, although it is an overly simplistic depiction of the complex relationship between neural function and moral judgment.

The continuous operation of sub-cortical affective systems, and their manner of negotiating with cognitive processes (Damasio, 2018), calls into question any attempt at imposing a dissociated and uncontaminated dichotomy between affect and cognition. There is an asymmetry where the former is always present in the latter, whereas the latter is not present in the former (Panksepp, 1998). Hence, we need a better way to conceptualize this relationship than the cognition-centric views that mainstream among neuroscientists today. Solms (2021) presents a theoretically plausible and empirically robust view that places *affect* at the center of human experiential self, consciousness, and comping in the world. By extension, we suggest that the qualitative and emotional character of morality has its roots in the affective structures of the brain stem. Human morality is a product of evolution, hence layered according to the layers of the brain, roughly as shown by the triune brain model (Panksepp, 1998). The more ancient parts are typically more fundamental.

Most neuroscientists agree that the central purpose of the human brain, as a product of evolutionary selection, has been to produce adequate adaptive responses to whatever the individual encounters in the environment as they relate to inner needs, notably homeostasis (Damasio, 2018, p. 44). Thus, the emerging insights from cognitive neuroscience point toward a model in which neural systems underlying automatic, intuitive, and emotional processes and controlled, deliberative, and cognitive processes are all involved in making moral judgments in order to respond appropriately to immediate circumstances. However, the complexity of how and when these parallel streams of processing act individually or in unison has been severely underestimated.

**Figure 1:**
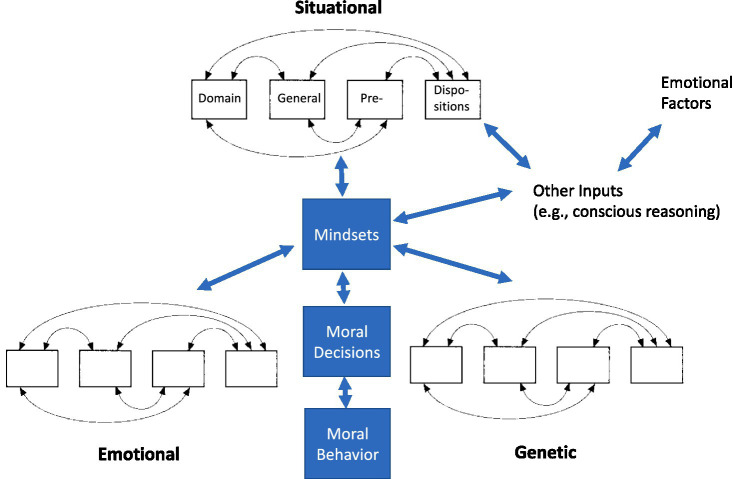
Hierarchy-herarchy model of moral behavior. A heterarchy contains elements (i.e., domain general pre-dispositions) connected to other elements in several ways (fully interconnected, circular, etc.). Individual heterarchies (associated with situational, emotional, genetic, and other factors) aggregate to form specific mindsets that are the unified response to a specific set of circumstances. We can express mindsets as moral decisions that may or may not have accompanied actions (moral behavior) associated with them.

*We see moral behavior as embodied in the interactive mechanisms of affective systems and social cognition and these mechanisms arising out of a set of domain-general primitive behavioral functions or predispositions that aggregate to produce increasingly complex behavior* (see Figure 1; Heyes, 2012; Heyes and Pearce, 2015).

These new, more complex predispositions can become more and more adapted to “social” problems and imbued with moral valence. Thus, creating a variety of mindsets, i.e., a mindset being a set of predispositions associated with specific social cognition and hence moral behavior. We further contend that moral behavior is conceived logically and functionally as organized drawing on insights from two different accounts of consciousness, respectively Dietrich (2003) and Solms (2021), as well as Pineda et al. (2018) definition of social behavior. The entire set of potential mindsets created is structurally hierarchical but heterarchically embedded (see Bruni and Giorgi, 2015), where activity is dynamically and contextually dependent.

A general-purpose definition of *heterarchy* is an organization where the elements are unranked (non-hierarchical) or where they possess the potential to be related, control or are controlled by others, depending on situational requirements (Crumley, 1979, p. 144). This suggests that activity within a heterarchy can be dynamic, distributed, possesses flexibility, adaptability, and that the relationships between elements are characterized by multiple intricate linkages that create circular, rather than hierarchical connections that change as situations evolve (McCulloch, 1945). Ogilvy (2016) presented one of the simplest illustrations of heterarchy and circular logic as a game of rock paper scissors—in which rock beats scissors, which beats paper, which beats rock.

A heterarchy may be parallel to a hierarchy, subsumed into a hierarchy, or it may contain hierarchies; the two kinds of structure are not mutually exclusive. In fact, each level in a hierarchical system is composed of a potentially heterarchical group which contains its constituent elements. In particular, heterarchical elements may be part of a *nested* hierarchical structure (Panksepp, 2011), in which higher structures depend on the function of structures below. For instance, studies show that if even a small segment of the brain stem is severed, immediate death follows. Whereas humans (with hydroencephalic kids) and other animals will continue to live and even interact in appropriate (albeit compromised) ways with the environment *entirely without cortical structures.* They will successfully navigate around obstacles, as long as the connection of optical nerves to the superior colliculi of the brain stem is intact; decorticate mice will, for example, more often than not, successfully raise a litter (Solms, 2021).

In a hierarchy–heterarchy, higher order structures perform increasingly more complex integrative functions and contribute more sophisticated and domain-specific content. A heterarchical organization means that the functional flow of information allows for the possibility of self-referentiality or recursiveness, reentrance, coordination of qualitatively different domains, and second-order-emergence (Bruni and Giorgi, 2015). This involves systems dynamically gained through experience, which are not pre-specified or determined *a priori* (Edelman and Gally, 2013).

In sum, we argue (with Narvaez, 2014) that understanding the neural basis of ethical behavior requires a set of principles that place social cognition, from which morality arises, in a framework that can help simplify and explain its evolution, development, and organization as *adaptive* behavior. Underlying this is the assumption that social cognition is not simply a high-level hallmark of human behavior but may be a basic, default mode of processing—one deeply embedded in our biology (Hari et al., 2015; Pineda et al., 2018). Brain structure–function relationships evolved phylogenetically and ontogenetically to produce networks capable of reliable computational performance that were at once adaptive to changing circumstances.

Such cognitive architectures maintain consistent, recognizable, and reproducible responses across individuals and yet keep many additional degrees of freedom for context-, stimulus- and task-dependent reconfiguration (Petersen and Sporns, 2015). Different configurations or mindsets of network activity make unique contributions—some involved in domain-general moral functions, others more engaged in domain-specific moral behaviors. The view that emerges is one in which elementary building blocks of social cognitive architecture, the aforementioned predispositions, coalesce into ever more complicated processes as a function of circumstances and challenges. These dynamically determined response mindsets are hierarchically and heterarchically organized, interdependent, and interpenetrating. The most sophisticated aspects are localized in higher order structures in the frontal cortex, but modulated and influenced by lower-level representations, and simultaneously imbued with emotional/moral valence. We believe that this conceptualization, which is an elaboration of something proposed by several investigators, including most recently Happé et al. (2017), could be pragmatic in organizing moral functions into a rational, phylogenetically and ontogenetically consistent approach. As Paul Nunez has expressed in his book, The New Science of Consciousness (Nunez, 2016), “consciousness is rooted in the dynamic patterns of multiple interacting scales.” We can say the same for social cognition and its expression of moral behavior, including beguiling mechanisms and deceptiveness with charming seduction for diversion.

## 3 Discussion—deception and beguiling mechanisms

In the film *Beguiled* (2017)—directed by Sofia Coppola and starring Colin Farrell, Nicole Kidman, Kirsten Dunst, Elle Fanning—a wounded Union soldier at a girls’ school in Virginia leads to jealousy, deception, self-deception and arousal of charm and deception in tandem. When looking at a text with a fact-oriented presentation—Plot: Beguiled. 2017^3^—it is difficult to get hold of subtle schemes, where charm and deception, as conscious/intentional and as unconscious deception, occur in an interaction between participants. Based on this text, a reader may get a vague idea of the thriller and its context, but for a viewer of the film, the subtlety of seductive and beguiling mechanisms plays out less unobtrusively between the participants.

In this theoretic paper, we focus on a mechanism that comprises two elements that often occur in tandem in episodes of personal interaction in everyday life—charm and deception. Our goal is to provide an easier understanding of “a rational psychology of deception.” We refer to it as “beguiling mechanisms.” When setting out with charm, deception it is more likely to come through. While we wrap deception in charm, it is a subtle and double outsmarting mechanism that might create alternative mindsets by overruling a calculative mindset with emotion, and then, deceptively, overrule emotion with a calculative mindset.

By starting out with charm to give a good impression, deception might pass with little interest in deliberation when deceptively overruling emotion as an integral part of moral judgment with a calculative mindset. Appeals to a shared sense of common identity and egalitarian fairness could trump considerations of economic efficiency and competition (a case of emotion overruling calculation); appeals to the rationality of behaving selfishly could trump initiatives to cooperate (calculation overriding emotion). Consider for example former US president George W. Bush’s remarkable appraisal of Russian president Vladimir Putin in 2001 (*The Slovenia Summit*): “I looked the man in the eye. I found him to be very straightforward and trustworthy.” Letting oneself be charmed may well be an effective and helpful psychological adaptation, where it is part of the sociality necessary for mutual trust and reciprocal cooperation to get off the ground (Trivers, 1971). However, this social predisposition, is on our analysis accompanied by the inherent risk of deceptive counterstrategies that have co-evolved with charm receptiveness. We suggest these mechanisms correspond to dynamic cognitive-affective interaction undergirded by the cooperation of discrete, but intimately interacting, decision systems.

Moral mindsets, on our account, complexly combine affective and cognitive processes. Cognitive processes, for instance, allow us to hold things in mind as objects and place them in relation to each other, allowing reasoning about the world to take place; while affective processes, meanwhile, assign these mental objects with meaning and qualitative distinctiveness. These interacting affective and cognitive processes represent the interplay between different levels of consciousness (hierarchically), as well as some degree of hemispherical specialization (laterally); the right hemisphere attuned more to the whole and meaning, the left hemisphere preoccupied more with detail, categories, and calculation (McGilchrist, 2009; Narvaez, 2014).

Solms (2021) argues that affect, or feeling, is the foundational form of consciousness. Affect, thus understood, is intrinsically conscious. Solms holds that one of Sigmund Freud’s important insights about the human mind was that mental processes are not intrinsically conscious. Foreshadowing the recent work of John Bargh and others, Freud suggested that most things go on in the mind without consciousness; we have the conscious mind, but also the workings of the “pre-conscious” and the “non-conscious” realms of the mind. Solms (2021) points out that this cannot apply to those mental processes that are affective, where they are directly felt and present in consciousness. In contrast, cognitive processes of a wide variety of sorts can operate unconsciously. Supporting this position, Kihlstrom (1996) presents evidence that perception can take place with no awareness of what we perceive, and learning without awareness of what we learn. Taking stock of accumulated neurological knowledge, these observations are uncontroversial. However, they bring out a sharp contrast between affect, which is *inherently* conscious and cognition that is *not*. As Solms (2021) points out, cognition may well be *inherently unconscious* unless it is *rendered* conscious, and (in line with Jaak Panksepp) the sources of this consciousness are emotions and other affects.

Affective processes have an important function for our ability to cope in the world. Latent hard-wired emotional response programs trigger some of the affective processes—stereotypical behavioral response patterns instilled in us by natural selection on an evolutionary time scale. Panksepp (1998) presents seven primary emotional systems in neuroanatomic and neurochemical detail, all of which are sub-cortically based (the fundamental emotional systems he lists still stand up to empirical scrutiny but may not be exclusive). Beyond primary process emotional systems, Solms (2021) suggests that affect allows us to respond to unexpected and unambiguous events where coping raises to a certain level of importance (for example, for survival). These are the specific events that warrant special attention, such as the tough decisions we face. We note that this is characteristic of moral decisions. If Solms is on the right track, we would expect the involvement of affective processes to be crucial for responses to moral dilemmas. We should also expect things to capture our attention in a manner that elicits desire (or the opposite) and prompt the need to make moral decisions (“should I invest time and resources to pursue this desire-inducing option or refrain from doing so?”).

Solms (2021) proposes that affects are functionally vital for our continued survival. He points out several examples that make this point clear. For example, the feeling of thirstiness prompts us to drink; sleepiness calls upon us to get some much-needed sleep. Our lives are, in this way, a constant struggle against the forces of entropy.

Morality, it should be noted, is not an exception. Human beings are vulnerable as individuals, and our coping well with nature, let alone thriving, depends on our comping well in the social domain (i.a., Boyd, 2018, Ch. 1). Crucially, the affective infrastructure of the human brain, as seen in other mammals, is evolved for social interdependencies and moral interaction in order to meet needs and survive, in the context of caring social community (Narvaez and Bradshaw, 2023). Solms (2021) presents a picture where affective states of pleasure and calm steadiness signify wellbeing and keep going to us. Physiologically this involves the typical “rest” and “digest” states, often intertwined with psychosociobiological activation of the care system in a social setting (Narvaez, 2014), on our account accompanied by specific episodes of moral interaction where “charm” plays a part.

Meanwhile, “unpleasure” (including, but broader than pain) and surprises alert the mind in ways that require specific types of action. Thus initiating stereotypical behavioral repertoires under the further guidance of corresponding affective feelings that sometimes allow for more calibrated ways of coping. The overall aim is to get back on track to a situation consistent with our continued survival (Solms, 2021). In this domain we have responses such as “fight”/anger, “flee”/fear, “freeze”/fear, “faint”/fear, “find”/panic (Panksepp, 1998), where the fear-based “flee” option, in a downregulated version, most closely aligns with the anxiety of possible “deception” on our account.

A key contribution in introducing beguiling mechanisms to the moral story is to highlight how positive and negative affect, interacting with percepts of social cognition, coexist to the point of fusing in certain important forms of moral interaction. This *affective fusion* reflects the ambiguity inherent in social situations that are characteristically moral. Thus, whereas primary level emotions are clearly valenced in the sense that they either are of a kind that feel “good” or “bad” (Panksepp, 1998), beguiling mechanisms give rise to mixed, conflicted, and unresolved feelings.

Affective feelings play a central role in practical moral life. Failing to act on the demands affective feelings place on us is a sure way toward death, disease, and demise (Solms, 2021). For example, failing to heed fear, we might fall off of some high place and suffer crippling or even fatal consequences. Too much fear can also be crippling and prevent successful coping in life, which is why evolution has selected for positive motivating emotions that many times can overcome fear. And hence the guidance of these positive emotions needs to be heeded as well. Each emotion is in this sense a decision system that jostles for attention. In beguiling mechanisms, we have an “edgy” decisions system that is presumably evolved to deal with the specific ambiguities we find in certain patterns of moral interaction. It gives the moral agent a way to enter open doors of sociality while leaving the door open (when possible) for possible escape (when needed).

Both cognitive and affecting processes work closely together in beguiling mechanisms. While affects represent exercises of allostasis (Sterling and Eyer, 1988), extending the reach meeting homoeotic needs, cognitive abilities further extend their reach, even allowing us to take action that involves temporal narratives and planning across time. This includes the characteristically human mental abilities of “mental time travel” (Suddendorf and Corballis, 1997), that enables us to place ourselves and others as objects in the past or into a projection of an uncertain the future. The anxieties and fears of deception issued by beguiling mechanisms are therefore not merely related to immediate social situations, but also to our moral imaginations and visions of a future where further social interaction could take place (Narvaez, 2016). Extended temporal aspects of beguiling mechanisms show that they have an especially wide-reaching functionality in human morality (beyond that of other living primates). The mechanisms themselves, however, carry evolutionary functionalities that we share with many other social animals (a point we return to below). This is likely a central component of the morality of other primates, alongside features like care, empathy, reciprocity, and even fairness (Waal, 1996). Thus, beguiling mechanisms in humans straddle across the main functional domains of human morality, namely protectionism (safety concerns), engagement (care and sociality), and imagination (intellectual growth; Narvaez, 2016).

Beguiling mechanisms refer to a complex nexus of affective and cognitive processes in the moral social-relation space of coping. There is an element of *competition*: who gets what from whom and at what cost? But there is also an element of *cooperation*; social interaction, including the more specific forms we find exemplified by social boding and commitment, may make in necessary for us to let ourselves be deceived—and furthermore self-deceived–to remain hopeful, even optimistic faced with adversity. All solitary options are worse; worse for us as individuals because we are the type of social, bonding creatures we are, but more fundamentally, worse in the evolutionary sense of maximizing the likelihood of the perpetuation of our species. The duality of beguiling mechanisms is important to grasp., and how they relate to the ambiguity of moral situations. Consider, for example, a marriage proposal: there will typically be emotions of charm and associated opportunities of cooperation at the receiving end, but simultaneously various fears of deception, conflicts, and opportunity losses.

There are several types of social and manipulative mechanisms that can influence a moral mindset, and we will focus on the ones we call “beguiling mechanisms.” Beguiling mechanisms is a composition of terms constructed for this cross-disciplinary paper in which we aim at a better understanding of how these mechanisms influence moral sensitivity, including a judgment of moral dilemmas, and the awareness of moral behavior. We consider, in this context, facets and dimensions of morality in mental processes, including the relation between the feeling of emotions and their underlying neural mechanisms. To reflect the complexity of the neural basis of moral judgment *in situ*, we propose a neurally inspired model, and have selected a combination of terms defining behavior that is wide rather than narrow. In describing beguiling mechanisms, we also refer to the importance of the embodied aspect of emotions (we see their primary function as the role of communicating closely with the body; Damasio, 2021). By embodiment, we mean thoughts, feelings, and behaviors grounded in bodily interaction with the environment (Damasio, 1999).

This and other social behaviors may all have evolved from a more basic sexual selection mechanism (Miller, 2007). Charles Darwin was the first to theorize that adornments distinguishing male animals from females evolved through sexual selection (see Figure 1). This is the process by which a female, seeking a mate for her offspring, chooses a male that flaunts the most colorful adornment, or shows the most impressive physique, or gives the appearance of being a good provider. Or that has, as Darwin (1871) said, “the power to charm the female.” Of course, selection will not only depend on what *is* being selected but also the ensuing reproductive success relatively speaking, or lack thereof, of that which is selected. Beguiling mechanisms, in this light, may be evolutionary old and serve a central function in reproductive success. We argue that this old set of mechanisms has been *repurposed* and *extended* so as to serve as a key component of our ability to cope in moral interactions.

We note that, “natural selection favors individuals who successfully manipulate the behavior of other individuals, whether or not this is to the advantage of the manipulated individuals” (Dawkins and Krebs, 1978, 309). However, moral interactions sometimes represent opportunities for locking in *mutual advantage*, at the *social* level. Beguiling mechanisms at play in human morality involves coping with the duality opportunities for mutual advantage and for falling prey to deception; between social engagement for mutual advantage and protection against loss of individual advantage. A socially nurturing social environment has been the standard condition in which human beings have evolved (Narvaez and Bradshaw, 2023). The “charm” aspect of beguiling mechanisms are therefore likely to be most central in early life years, while protection from “deception” is like to make its entry later as the stakes social interaction increases.

Fisher (1999) and Fisher (1930) extended Darwin by proposing that run-away sexual selection can take place because females choose the most outstanding mates on attractiveness features such as wing length. And that this is a process that can proceed far beyond the original adaptive functionality for that feature, where females primarily select on specific physical attributes within a relatively narrow window of attention confined to discrete episodes of social interaction. From the viewpoint of long-term adaptation, selective processes can go regressively off track in periods before selective pressures steer them onto more adaptively efficient paths (if at all). At all times, there will be an important exchange between two parties taking place in these selective processes.

**Figure 2:**
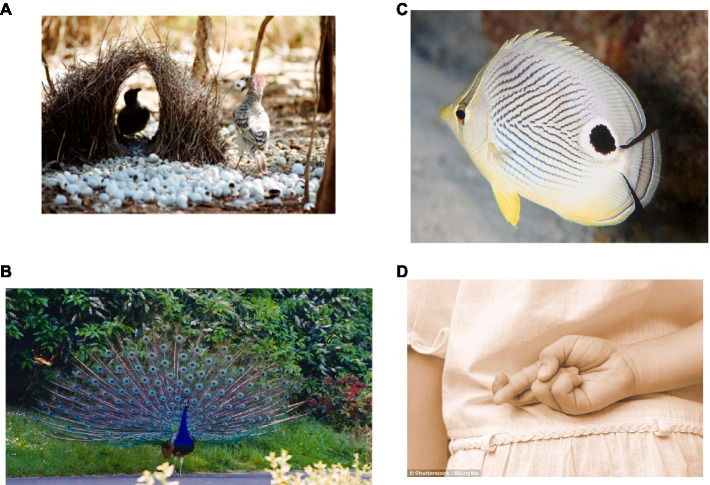
Examples of deception and beguiling mechanisms in social behavior. Such behaviors have become part and parcel of nature and intrinsic and necessary features of human existence. **(A)** Bowerbird male, well known for making elaborate constructions, lavished with decorative objects, trying to impress and attract a mate. John Hill/Wikimedia reproduced under the terms of CC-BY-SA 3.0 **(B)** Male peacock performing specialized copulation calls without the presence of females, considered dishonest signals of male mating attempts. Dineshkannambadi/Wikipedia reproduced under the terms of CC-BY-SA 3.0. **(C)** Four-eye butterflyfish. Many fish and insects sport a large eye spot somewhere on their body to throw off predators. LASZLO ILYES/Flickr reproduced under the terms of CC-BY 2.0. **(D)** Child with crossing fingers. The ability of humans to lie and engage in other forms of deception is a source of great social power, as it allows people to shape interactions in ways that serve their interests BlurryMe/Shutterstock.

Weldon and Burghardt (1984) expanded on these ideas and advanced the notion of “deception divergence.” They argued that as males of the same species competed for female favors, employing deception, guile, and mimicry, there was competition between the dominant “honest” signalers and the “dishonest” inferior, less established, or younger males who mimicked them. This behavior led to a kind of signaling arms race with both the “honest” and “dishonest” animals, slowly gaining more and more exaggerated traits functionally suited to outdo their competitors. This could be the case if within-group competition in a specie is more important for survival than the competitive pressure between species. As a result, male adornment, along with increasingly deceitful behavior, became more and more colorful and bizarre over thousands of generations. Hence, beguiling mechanisms, along with self-deception and mimicry, play an inordinately important role in sexual selection among the same species. By unconsciously deceiving oneself, an animal might likewise become a more effective deceiver of others (Greenwald, 1988). In some fish, a male gains access to a nest defended by a resident male when mimicking the behavior of a receptive female by releasing his sperm to fertilize the eggs (Lockard, 1988). Maybe humans learn from being deceived and become more suspicious, whereas they are easily persuaded by charming seduction. Such behaviors become part and parcel of nature and intrinsic and necessary features of human existence (Figure 2).

With this backdrop from the general picture of the animal kingdom, we will now inquire specifically into corresponding episodes involving human decision-making. A neurological example of this dissociation involves patients with lesions in dorsolateral prefrontal cortex, who are incapable of behaving socially, albeit their judgment and understanding is unaffected (Anderson et al., 1999). There is support for the claim that decisions and choices influenced by context and by unconscious systems. They do so by directing behavior before the decision maker is even aware of making a choice (by several hundred milliseconds), e.g., as measured by EEG (Libet et al., 1983). Hence, we support the premise that moral intuition develops before conscious reasoning (Haidt, 2013). However, we also believe that manipulation of moral intuition is a strongly influential part of everyday behavior, including maneuvering and circumventing awareness, to produce alternative mindsets critical for making judgments and moral decisions.

## 4 Conclusion and implications

The theoretical argument we have sketched in this paper has some similarities to what Moll and colleagues proposed in 2008. In their Motivational Approach, they argued that social behavior (or morality) is determined by biological predispositions (reflected in intuitions) that trigger moral emotions and these modulate behaviors. Similar to our argument, Moll et al. suggested that such predispositions evolved during human evolution as motivational forces to foster prosocial behavior. Haidt and Graham (2007) named several such basic predispositions, which, through a complex interplay of social, cognitive, and emotional processes, condition human moral judgments and behavior. Our proposal differs in providing a neurally inspired theoretical framework for the taxonomy and interrelationships of these predispositions to create distinct mindsets. We have argued that moral mindsets are situation- and context-specific and therefore require a dynamic, hierarchical-heterarchical perspective to understand their root basis. In such a dynamic model, factors such as emotion, self-sacrifice, and embodied aspects of behavior, such as deception, play an understated but critical role. These predispositions can become more and more adapted to solving social” problems imbued with moral valence, thus creating a moral mindset.

To make the case, we have highlighted one such example of an activity that creates problems for traditional explanations of ethical behavior. Human beings are social, with certain needs that can only be satisfied in the social world. During the Phanerozoic era, we evolved complex means to interact with others, including the ability to deceive and more subtly to deceive while charming the other. Humans sometimes learn valuable lessons from being deceived—when affective response raises deception to the level of associative learning or even further to the level of conscious awareness—allowing suspicion to enter the realm of moral choice. The flexible capacity to continue to be swayed by charming seduction—the beguiling mechanisms—may be kept intact, allowing for opportunities for future social interaction, trust, and potentially fruitful modes of cooperation and group cohesion.

Moral behavior has traditionally, under the guiding lights of prominent philosophers, been conceptualized as rational thinking. However, it is now recognized that emotions play as key a role in moral decision-making. In particular, the subjective feeling component of emotions allows decision-makers to *feel* their way through difficult moral decisions. Moral decisions therefore involve a dynamic interplay between affective and cognitive brain systems, where internal emotions connect objects in the outside world—as in “I feel like this about that”—with a dynamic hierarchy of internal needs that initially prompt these affective feelings. Grounding emotion and rationality and connecting them to ethical behavior has been problematic. This is because the human mind is a product of evolution. Meaning that morality may be grounded in our perceptions and embodied representations of the world in which we live, and the various needs of the body, which when urgent, show up as distinctive affective feelings and associated stereotypical behavior. We believe that further research should emphasize empirical ethics, to gain more insight into beguiling mechanisms and ethics by gathering data about charm along with sophisticated manipulation (*cf.* the plot: beguiled 2017 below). We will explore how easily people are diverted from their own plans and beliefs, if they are persuaded with charm rather than experimenter’s pressure and discontent.

## Data availability statement

The original contributions presented in the study are included in the article/supplementary material, further inquiries can be directed to the corresponding author.

## Author contributions

HK, JP, and SW: substantial contributions to conception and design, content analysis, and interpretation. SW: literature search and preparation of relevant literature. JP: drafting the article or revising it critically for important intellectual content. HK and JP: final approval of the version to be published. All authors contributed to the article and approved the submitted version.

## Footnotes

^1^Research suggests that effortful cognitive processes, such as those underpinning calculation, frequently suppress emotions (Panksepp, 1998). Similarly, Crowding Out theory (Sandel, 2012) points out that when people perceive money as extrinsic motivation, a calculative mindset activates by suppressing emotional influence, i.e., disinhibiting unethical behaviors crowds out intrinsic motivation (Sandel, 2012, 19). When framing a task as a decision between two alternatives, as opposed to asking for an emotional reaction, the subject appears absorbed in an analytical process that is apt to induce unethical behavior. Studies in experimental psychology suggest that such framing effects can be strong, persistent, and, in certain settings, manipulatable by small tweaks (Knobe, 2003). This is the case with so-called ‘the Knobe effect’, where a statement is altered (e.g., “help the environment” vs. “harm the environment”) so that only its *presentation* differs, while keeping all facts of the decision the same. Merely tweaking the manner of presentation in these cases reliably shifts the relative evaluation of the options presented in the decision problems.

^2^Semantically, there is a line connecting the term beguiling to “fool” and to “charm.” For the first term, the connotation is to “deceive,” “mislead,” “outwit,” “fake out,” “outdo,” “outfox,” “outmaneuver,” “outsmart,” “circumvent,” “bypass” and “dodge.” As for the second term, there is a connotation to “amuse,” “entice” and “seduce.” In our current sketch of deception as *charming seduction* or beguiling mechanism, we have developed theoretical assumptions about the ontology and purpose of such mechanisms. Panksepp (1998), Damasio (1999), and Greene et al. (2001), among others, suggest that emotions play an integral role in moral judgments.

^3^ For the plot of Beguiled see https://en.wikipedia.org/wiki/The_Beguiled_(2017_film).

## References

[ref1] Anderson S. W. Bechara A. Damasio H. Tranel D. Damasio A. R. (1999). Impairment of social and moral behavior related to early damage in human prefrontal cortex. Nat. Neurosci. 2, 1032–1037. doi: 10.1038/14833 10526345

[ref3] Bargh J. A. (1994). “The four horsemen of automaticity: intention, awareness, efficiency, and control as separate issues” in Handbook of social cognition. eds. Wyer R. Srull T. (New Jersey: Lawrence Erlbaum), 1–40.

[ref4] Bargh J. A. (2017). Before you know it, the unconscious reasons we do what we do: Touchstone.

[ref5] Bechtel W. (2008). Mental mechanisms: Philosophical perspectives on cognitive neuroscience. New York: Routledge.

[ref6] Boyd R. (2018). A different kind of animal: How culture transformed our species. Princeton: Princeton University Press.

[ref7] Brand C. (2016). Dual-process theories in moral psychology: Interdisciplinary approaches to theoretical, empirical and practical considerations. Wiesbaden: Wiesbaden: Springer Fachmedien Wiesbaden GmbH.

[ref8] Bruni L. E. Giorgi F. (2015). Towards a heterarchical approach to biology and cognition. Prog. Biophys. Mol. Biol. 119, 481–492. doi: 10.1016/j.pbiomolbio.2015.07.005 26236011

[ref10] Crumley C. L. (1979). Three locational models: an epistemological assessment for anthropology and archaeology. Adv. Archeol. Method Theory 2, 141–173.

[ref9001] Damasio A. (1999). The feeling of what happens: Body and emotion in the making of consciousness. New York: Harcourt College Publishers.

[ref11] Damasio A. (2018). The strange order of things: Life, feeling, and the making of cultures. Westminster: Knopf Doubleday Publishing Group.

[ref12] Damasio A. (2021). Feeling & knowing: Making minds conscious. New York: Patheon Books. 10.1080/17588928.2020.1846027 33323038

[ref13] Darwin C. (1871). The descent of man, and selection in relation to sex. London: Princeton University Press.

[ref14] Dawkins R. Krebs J. R. (1978). “Animal signals: information or manipulation” in Behavioralecology: An evolutionary approach. eds. Krebs J. R. Davies N. B. (Oxford, England: Blackwell), 282–309.

[ref17] Dietrich A. (2003). Functional neuroanatomy of altered states of consciousness: the transient hypofrontality hypothesis. Conscious. Cogn. 12, 231–256. doi: 10.1016/s1053-8100(02)00046-6 12763007

[ref18] Edelman G. M. Gally J. A. (2013). Reentry: a key mechanism for integration of brain function. Front. Integr. Neurosci. 7:63. doi: 10.3389/fnint.2013.00063 23986665 PMC3753453

[ref19] Fisher R. A. (1930). The Genetical theory of natural selection. Oxford: Clarendon Pr.

[ref20] Fisher R. A. (1999). The genetical theory of natural selection: A complete variorum edition. Oxford: Oxford University Press.

[ref22] Gopnik A. (2016). The gardener and the carpenter: What the new science of child development tells us about the relationship between parents and children. 1 ed.. New York: Farrar, Straus and Giroux.

[ref26] Greene J. D. Morelli S. A. Lowenberg K. Nystrom L. E. Cohen J. D. (2008). Cognitive load selectively interferes with utilitarian moral judgment. Cognition 107, 1144–1154. doi: 10.1016/j.cognition.2007.11.004, PMID: 18158145 PMC2429958

[ref27] Greene J. D. Nystrom L. E. Engell A. D. Darley J. M. Cohen J. D. (2004). The neural bases of cognitive conflict and control in moral judgment. Neuron 44, 389–400. doi: 10.1016/j.neuron.2004.09.027, PMID: 15473975

[ref28] Greene J. D. Sommerville R. B. Nystrom L. E. Darley J. M. Cohen J. D. (2001). An fMRI investigation of emotional engagement in moral judgment. Science 293, 2105–2108. doi: 10.1126/science.1062872 11557895

[ref9002] Greenwald A. G. (1988). “Self-knowledge and self-deception,” in: Self-deception: An adaptive mechanism? eds. Lockard J. S. Paulhus D. L. (Englewood Cliffs, NJ: Prentice-Hall), pp. 113–131.

[ref29] Haidt J. (2001). The emotional dog and its rational tail: a social intuitionist approach to moral judgment. Psychol. Rev. 108, 814–834. doi: 10.1037/0033-295X.108.4.814, PMID: 11699120

[ref30] Haidt J. (2013). Moral psychology for the twenty-first century. J. Moral Educ. 42, 281–297. doi: 10.1080/03057240.2013.817327

[ref31] Haidt J. Graham J. (2007). When morality opposes justice: conservatives have moral intuitions that liberals may not recognize. Soc. Justice Res 20, 98–116. doi: 10.1007/s11211-007-0034-z

[ref32] Happé F. Cook J. L. Bird G. (2017). The structure of social cognition: in(ter)dependence of Sociocognitive processes. Annu. Rev. Psychol. 68, 243–267. doi: 10.1146/annurev-psych-010416-044046, PMID: 27687121

[ref33] Hari R. Henriksson L. Malinen S. Parkkonen L. (2015). Centrality of social interaction in human brain function. Neuron 88, 181–193. doi: 10.1016/j.neuron.2015.09.022, PMID: 26447580

[ref34] Heyes C. (2012). What's social about social learning? J. comparative psychol. (1983) 126, 193–202. doi: 10.1037/a0025180 21895355

[ref35] Heyes C. Pearce J. M. (2015). Not-so-social learning strategies. Proc. Biol. Sci. 282:20141709. doi: 10.1098/rspb.2014.1709, PMID: 25608880 PMC4344138

[ref37] Kahane G. Wiech K. Shackel N. Farias M. Savulescu J. Tracey I. (2012). The neural basis of intuitive and counterintuitive moral judgment. Soc. Cogn. Affect. Neurosci. 7, 393–402. doi: 10.1093/scan/nsr005, PMID: 21421730 PMC3324565

[ref9003] Kihlstrom J. (1996). “Perception without awareness of what is perceived, learning without awareness of what is learned,” in The Science of Consciousness: Psychological, Neuropsychological and Clinical Reviews. ed. Velmans M. (London: Routledge), pp. 23–46.

[ref40] Knobe J. (2003). Intentional action and side effects in ordinary language. Analysis 63, 190–194. doi: 10.1111/1467-8284.00419

[ref41] Koenigs M. Young L. Adolphs R. Tranel D. Cushman F. Hauser M.. (2007). Damage to the prefrontal cortex increases utilitarian moral judgements. Nature (London) 446, 908–911. doi: 10.1038/nature05631, PMID: 17377536 PMC2244801

[ref42] Kohlberg L. (1969). “Stage and sequence: the cognitive-developmental approach to socialization” in Handbook of socialization theory and research. ed. Goslin D. A. (Chicago, IL: Rand McNally), 347–480.

[ref43] Libet B. Gleason C. A. Wright E. W. Pearl D. K. (1983). Time of conscious intention to act in relation to onset of cerebral activity (readiness-potential). Brain 106, 623–642. doi: 10.1093/brain/106.3.623, PMID: 6640273

[ref9004] Lockard J. S. (1988). “Origins of Self-Deception: Is lying to oneself uniquely human?,” in Self-deception: An adaptive mechanism? eds. Lockard J. S. Paulhus D. L. (Englewood Cliffs, NJ: Prentice-Hall), pp. 14–22.

[ref44] McCulloch W. S. (1945). A heterarchy of values determined by the topology of nervous nets. Bull. Math. Biophys. 7, 89–93. doi: 10.1007/BF02478457 21006853

[ref45] McGilchrist I. (2009). The master and his emissary. New Haven: Yale University Press

[ref46] Miller G. F. (2007). Sexual selection for moral virtues. Q. Rev. Biol. 82, 97–125. doi: 10.1086/517857 17583267

[ref47] Moll J. Oliveira-Souza R. D. Garrido G. J. Bramati I. E. Caparelli-Daquer E. M. Paiva M. L.. (2007). The self as a moral agent: linking the neural bases of social agency and moral sensitivity. Soc. Neurosci. 2, 336–352. doi: 10.1080/17470910701392024, PMID: 18633822

[ref48] Narvaez D. (2014). Neurobiology and the development of human morality: Evolution, culture, and wisdom (Norton Series on Interpersonal Neurobiology). New York: WW Norton & Company.

[ref49] Narvaez D. (2016). Embodied morality: Protectionism, engagement and imagination. London: Palgrave Pivot London.

[ref50] Narvaez D. Bradshaw G. A. (2023). The evolved nest: Nature's way of raising children and creating connected communities. Berkeley: North Atlantic Books.

[ref52] Nunez P. L. (2016). The new science of consciousness: Exploring the complexity of brain, mind, and self. New York: Prometheus Books.

[ref53] Ogilvy J. (2016). Heterarchy: An idea finally ripe for its time. Austin: Stratfor/Worldview.

[ref54] Panksepp J. (1998). The periconscious substrates of consciousness: affective states and the evolutionary origins of the self. J. Conscious. Stud. 5, 566–582.

[ref55] Panksepp J. (2011). Cross-species affective neuroscience decoding of the primal affective experiences of humans and related animals. PLoS One 6, 1–15. doi: 10.1371/journal.pone.0021236 PMC3168430 21915252

[ref57] Petersen S. E. Sporns O. (2015). Brain networks and cognitive architectures. Neuron 88, 207–219. doi: 10.1016/j.neuron.2015.09.027, PMID: 26447582 PMC4598639

[ref58] Pfaff D. W. (2015). The altruistic brain: How we are naturally good. Oxford: Oxford: Oxford University Press, Incorporated.

[ref59] Piaget J. (1932). The moral judgment of the child (Vol. 85). London: Routledge & Kegan Paul.

[ref60] Pineda J. A. Singh F. Chepak K. (2018). “The language and structure of social cognition: an integrative process of becoming the other” in The neuroscience of empathy, compassion, and self-compassion. eds. Stevens L. C. Woodruff C. C. (Netherland: Elsevier), 267–283.

[ref62] Rand D. G. (2016). Cooperation, fast and slow. Psychol. Sci. 27, 1192–1206. doi: 10.1177/0956797616654455 27422875

[ref63] Rand D. G. Kraft-Todd G. T. (2014). Reflection does not undermine self-interested prosociality. Front. Behav. Neurosci. 8:300. doi: 10.3389/fnbeh.2014.00300 25232309 PMC4153292

[ref64] Redish A. D. (2013). The mind within the brain: How we make decisions and how those decisions go wrong. New York: Oxford University Press.

[ref66] Sandel M. J. (2012). What money can't buy: The moral limits of markets. London: Penguin Books.

[ref68] Solms M. (2021). The hidden spring: A journey to the source of consciousness. London: Profile Books Ltd.

[ref9005] Sterling P. Eyer J. (1988). “Allostasis: A new paradigm to explain arousal pathology,” in Handbook of life stress, cognition and health. eds. Fisher S. Reason J. (John Wiley & Sons), pp. 629–649.

[ref69] Suddendorf T. Corballis M. C. (1997). Mental time travel and the evolution of the human mind. Genet. Soc. Gen. Psychol. Monogr. 123, 133–167. PMID: 9204544

[ref74] Trivers R. L. (1971). The evolution of reciprocial altruism. Q. Rev. Biol. 46, 35–57. doi: 10.1086/406755

[ref77] Waal F. D. (1996). Good natured: The origins of right and wrong in humans and other animals. Cambridge, Mass: Harvard University Press.

[ref78] Waal F. D. (2016). Are we smart enough to know how smart animals are? New York:London: W. W. Norton & Company; Granta.

[ref79] Wang L. Zhong C.-B. Murnighan J. K. (2014). The social and ethical consequences of a calculative mindset. Organ. Behav. Hum. Decis. Process. 125, 39–49. doi: 10.1016/j.obhdp.2014.05.004

[ref80] Weldon P. J. Burghardt G. M. (1984). Deception divergence and sexual selection. Z. Tierpsychol. 65, 89–102. doi: 10.1111/j.1439-0310.1984.tb00092.x

[ref81] Wood W. (2019). Good habits, bad habits: The science of making positive changes that stick. New York: Farrar, Straus and Giroux.

[ref82] Zhong C.-B. (2011). The ethical dangers of deliberative decision making. Adm. Sci. Q. 56, 1–25. doi: 10.2189/asqu.2011.56.1.001

